# Tempo-spectral multiplexing in flow cytometry with lifetime detection using QD-encoded polymer beads

**DOI:** 10.1038/s41598-019-56938-2

**Published:** 2020-01-20

**Authors:** Daniel Kage, Katrin Hoffmann, Galina Nifontova, Victor Krivenkov, Alyona Sukhanova, Igor Nabiev, Ute Resch-Genger

**Affiliations:** 1 Federal Institute for Materials Research and Testing (BAM), Biophotonics Division 1.2, Richard-Willstätter-Str. 11, D-12489 Berlin, Germany; 2 Department of Physics, Humboldt-Universität zu Berlin, Newtonstr. 15, D-12489 Berlin, Germany; 3 Laboratory of Nano-bioengineering, National Research Nuclear University MEPhI (Moscow Engineering Physics Institute), 115409 Moscow, Russian Federation; 4 Laboratoire de Recherche en Nanosciences, LRN-EA4682, Université de Reims Champagne-Ardenne, 51100 Reims, France; 5 Sechenov First Moscow State Medical University, 119991 Moscow, Russian Federation

**Keywords:** High-throughput screening, Nanoscale materials

## Abstract

Semiconductor quantum dots (QDs) embedded into polymer microbeads are known to be very attractive emitters for spectral multiplexing and colour encoding. Their luminescence lifetimes or decay kinetics have been, however, rarely exploited as encoding parameter, although they cover time ranges which are not easily accessible with other luminophores. We demonstrate here the potential of QDs made from II/VI semiconductors with luminescence lifetimes of several 10 ns to expand the lifetime range of organic encoding luminophores in multiplexing applications using time-resolved flow cytometry (LT-FCM). For this purpose, two different types of QD-loaded beads were prepared and characterized by photoluminescence measurements on the ensemble level and by single-particle confocal laser scanning microscopy. Subsequently, these lifetime-encoded microbeads were combined with dye-encoded microparticles in systematic studies to demonstrate the potential of these QDs to increase the number of lifetime codes for lifetime multiplexing and combined multiplexing in the time and colour domain (tempo-spectral multiplexing). These studies were done with a recently developed novel luminescence lifetime flow cytometer (LT-FCM setup) operating in the time-domain, that presents an alternative to reports on phase-sensitive lifetime detection in flow cytometry.

## Introduction

Flow cytometry (FCM), where either single cells or particles are optically detected in a flow, is an established and widespread multiparametric fluorescence technique used for many routine and research applications in biology, medical diagnostics, and food analysis^1–5^. FCM typically relies on organic luminophores for optical encoding and multiplexing as well as analyte quantification^6–10^. State-of-the-art FCM instruments are designed to detect spectral and/or intensity codes and can distinguish a large number of different colour and intensity codes, e.g. in the case of bead-based assays^5,11–13^. Commonly applied spectral multiplexing approaches utilizing organic luminophores are, however, limited by the spectral overlap of the emission bands of the different encoding dyes which limits the number of distinguishable codes and can require signal compensation and cross talk correction^6,14,15^. The exploitation of the luminophore-characteristic luminescence lifetime (LT) as additional encoding parameter could in principle increase the number of accessible codes in flow cytometry analysis, thereby increasing the degree of multiplexing^6,7,14,16–21^. Time-resolved fluorescence measurements in flow cytometry and lifetime encoding have been discussed for decades^8,22–28^. The concept of lifetime multiplexing in a flow, however, has not been adopted in routine applications up to now due to the limited measurement time per object resulting in reduced photon count numbers^27,29^. Moreover, the vast majority of reports on lifetime detection in flow cytometry relies on frequency-domain techniques (phase fluorometry), which used to be less expensive as fast detectors required for photon counting-based detection in the time domain, and present specific applications^8,9,14,18,28,30–32^. This has been highlighted e.g., in a recently published comprehensive review article from J.P. Houston *et al*.^8^, revealing only a very small number of research articles related to cytometry studies in the time domain, mostly associated with microfluidics. Even though time-domain and frequency-domain flow cytometry systems are principally equivalent with respect to resolution, time-domain methods can be superior for low signal intensities due to their higher sensitivity^33^. Here, we focus on the evaluation of the potential of LT-FCM in the time-domain by systematic studies with lifetime-encoded microbeads using different types of encoding luminophores.

The time range accessible with beads encoded with organic luminophores is restricted to lifetimes <10 ns, which enables only the temporal distinction of very few lifetime codes for excitation with a single light source and detection within a fixed spectral window. Ideal colour barcodes are semiconductor quantum dots (QDs) due to their versatile excitability with virtually all wavelengths below the optical bandgap in conjunction with the relatively narrow and symmetrically shaped emission bands. Even though there are concerns about the environmental impact of the QDs, their very attractive optical-spectroscopic properties seem to outweigh this drawback for certain applications^34–36^. In the last decades, numerous examples for their use as optical reporters in spectrally encoding or multiplexing approaches in the life sciences have been reported, ranging from luminescence assays over immunohistochemistry to the flow cytometric detection of biomarkers in body liquids^35,37–39^. Their luminescence lifetimes have been, however, rarely exploited as encoding parameter^21,36^, although they cover a time range which is not accessible with other luminophores. This encouraged us to assess the potential of different types of QDs to expand the lifetime range of luminophores for the encoding of polymer carrier beads in time-resolved flow cytometry. For lifetime-encoding of microbeads, we used either the layer-by-layer (LbL) technique for QD-staining of polymer beads^40^, or applied PMMA beads stained with organic luminophores incorporated during particle synthesis^41^. The resulting dye-stained microspheres exhibit lifetimes in the lower nanosecond range^41^, e.g. below 10 ns, whereas the LbL-coated QD microbeads show fluorescence lifetimes in the order of >10 ns. The combination of both types of lifetime-encoded bead systems significantly expands the accessible lifetime range for multiplexing applications^42–44^. Finally, the detectability and distinction of multiple codes in a bead mixture was assessed using a unique (prototype) compact fluorescence lifetime flow cytometer (LT-FCM setup)^21^.

## Materials and Methods

### Materials

Two types of luminophore-encoded beads were used. The first type were QD-decorated beads based on carboxylated melamine formaldehyde beads purchased from Microparticles GmbH, Germany. These beads were used as templates for QD-encoding employing a polyelectrolyte-based layer-by-layer (LbL) deposition technology with oppositely charged poly(allylamine hydrochloride) (PAH) and poly(sodium 4-styrensulfonate) (PSS) polymers (Sigma-Aldrich) and electrostatic immobilization of water-soluble negatively charged QDs presented elsewhere (see also Fig. S2 of the Electronic Supplementary Information (ESI))^35^. We used CdSe(core)/ZnS(shell) QD500 or CdSe(core)/CdS/ZnS(multilayer shell) QD645 core/shell QDs with fluorescence maxima at 500 nm and 645 nm, respectively. The mean ζ-potential values of QD500 and QD645 were determined to (−24.7 ± 1.6) mV and (−29.2 ± 4.1) mV, respectively, and their mean hydrodynamic diameters were in the range of 15.2 nm to 28.3 nm (see Fig. S1 of the ESI). The quantum yields of the QDs used in encoding of the polymer beads were determined to be about 99% (QD500) and 40% (QD645). The polymer shell of the QD-encoded microparticles was formed according to the following scheme: melamine formaldehyde core/PAH/PSS/PAH/PSS/PAH/QDs/PAH/PSS/PAH/PSS/PAH/PSS with a final PSS layer.

Briefly, 0.5 mL of a 2 mg/mL PAH aqueous solution in 0.5 mol/L NaCl was added to 0.5 mL of a suspension containing ≈3.5 × 10^8^ carboxylated melamine formaldehyde microparticles. The suspension was sonicated in an ultrasound bath for a short period of time and incubated while shaking for 20 min. Excess polymer was removed by three washing and centrifugation cycles using ultrapure water. The resulting pellet of PAH-coated microbeads was resuspended in 0.5 mL of ultrapure water. Then, a PSS layer was deposited using 0.5 mL of a 2 mg/mL PSS dissolved in 0.5 mol/L NaCl aqueous solution under the same conditions. This procedure was repeated to sequentially apply the next PAH, PSS, and PAH layers with each layer deposition followed by three washing-centrifugation cycles. QD loading was achieved by incubating positively charged beads with the negatively charged QDs for 80 min while permanently shaking the reaction mixture.

The second type of luminophore-loaded beads were commercially available fluorescent poly(methyl methacrylate) (PMMA) beads received from PolyAn GmbH, Germany. These beads are stained with a single organic dye, here either with PolyAn Red5^45^ or with the widely used photostable dye rhodamine 6G (Rh6G)^46–48^. The spectroscopic properties of dye-stained PMMA beads have been extensively examined and previously published in a separate study^41^. The encoding dyes were chosen to be excitable at 488 nm and have emission maxima at 550 nm and 690 nm, respectively.

### Ensemble fluorescence measurements

Steady state photoluminescence measurements and measurements of the photoluminescence decay curves were carried out on a calibrated fluorometer FLS920 (Edinburgh Instruments). This instrument is equipped with a xenon lamp for continuous wave excitation and a pulsed Fianium SC400-2-PP supercontinuum laser (NKT Photonics A/S) for time-resolved studies. For the latter measurements, the repetition rate of the laser was set to 5 MHz. A fast R3809U-50 multichannel plate photomultiplier (MCP-PMT) detector (Hamamatsu Photonics K.K.) was used to collect the luminescence signals. The full width at half maximum (FWHM) of the instrument response function (IRF) for time-resolved measurements is around 250 ps. The spectral resolution of the instrument for the measurement of emission spectra is approximately 6 nm. The measured decay kinetics were evaluated using the reconvolution procedure of the FAST program (Edinburgh Instruments Ltd.). From the measured, multiexponential decays, the intensity-weighted average lifetimes τ_int_ were determined from the least-squares multiexponential decay fits to the data.

All optical-spectroscopic ensemble studies were performed at room temperature using 10 mm × 10 mm quartz cuvettes from Hellma GmbH. The bead samples were continuously stirred to prevent particle precipitation. Magic angle polarizer settings^49^ were applied to avoid measurement artefacts due to rotational diffusion.

### Confocal laser scanning microscopy (CLSM) and fluorescence lifetime imaging microscopy (FLIM)

Single-particle microscopic measurements were performed with the lifetime-encoded beads suspended in water and transferred onto a coverslip. The microscopy images were recorded with a FluoView FV1000 microscope (Olympus GmbH, Germany). A multiline argon ion laser (488 nm, 30 mW) was used as the excitation light source. The excitation light was reflected by a dichroic mirror DM405/488 and focused onto the sample through an Olympus objective UPLSAPO 60xW (numerical aperture N.A. 1.2). The emitted photons were collected with the same objective and detected with photomultiplier tubes (PMTs) in different spectral channels defined by grating monochromators and optical filters. Spatially resolved emission spectra were recorded using a beam splitter BS20/80, a spectral resolution of 5 nm, and a spectral step size of 2 nm. FLIM measurements were carried out with the FV1000 microscope equipped with a FLIM−fluorescence correlation spectroscopy upgrade kit (PicoQuant GmbH) using the same samples. The excitation light source was a diode laser (485 nm, 5 MHz repetition rate). A dichroic mirror (DM405/488) was used to reflect the excitation light onto the sample. The emission light was collected with a 525 nm long-pass filter and a single-photon avalanche diode (SPAD). Data acquisition and analysis of the luminescence decay curves were carried out with the TimeHarp 200 TCSPC PC board using SymPhoTime software (PicoQuant GmbH, Germany)^50^.

### Time-resolved (lifetime) flow cytometry (LT-FCM)

Flow cytometry measurements with ns time resolution were performed with a recently developed Quantum P/pantau flow cytometer (Quantum Analysis GmbH, Germany). This LT-FCM setup was recently described in detail^21^. The instrument is equipped with a square-wave-modulated laser diode emitting at 488 nm (Nichia Corporation, Japan) with a repetition rate of 5 MHz (average output power was 50 mW), one detector for time-resolved photon counting with a time binning of 2.5 ns as well as three conventional PMTs in low-bandwidth mode for the side-scattered light channel and two fluorescence channels. One of the steady-state detectors with a (520 ± 14) nm bandpass filter was used as the ‘green’ channel. A 530 nm longpass filter (LP) in front of the detector was employed for the time-resolved measurements. The luminescence lifetimes were determined from photoluminescence intensity decay curves of single beads by means of Eq. (1)^49^.

1 $${\tau }_{{\rm{mean}}}=\frac{{\int }_{0}^{\theta }tI(t){\rm{d}}t}{{\int }_{0}^{\theta }I(t){\rm{d}}t}\approx \frac{{\sum }_{j=1}^{{j}_{{\rm{\max }}}}{t}_{j}{I}_{j}}{{\sum }_{j=1}^{{j}_{{\rm{\max }}}}{I}_{j}}$$

In Eq. (1), $I(t)$ denotes luminescence intensity at time $t$ and $\theta$ is the upper integration limit. Strictly, this equation is only valid for $\theta \to \infty$. For discrete data, the integral has to be replaced by a sum with finite upper summation index and intensity values ${I}_{j}$ in discrete time bins with time *t*_*j*_. In the presented LT-FCM measurements, the lifetimes were determined for each single object.

## Results and Discussion

We used four different types of luminescent beads referred to as DyeG, DyeR, QDG, and QDR in the following. DyeG and DyeR contain photostable organic luminophores Rh6G^46–48^ and Red5, respectively, whereas codes QDG and QDR are loaded with QD500 and QD645. In the following, first the optical properties of these beads obtained by ensemble and microscopic photoluminescence measurements are briefly presented. Then, their applicability for time-resolved or lifetime flow cytometry (LT-FCM) is demonstrated.

### Optical properties of bead ensembles

**Figure 1:**
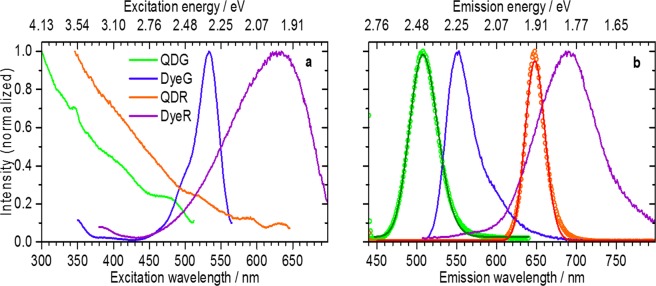
Photoluminescence spectra of ensembles of luminophore-loaded beads dispersed in water. (**a**) Photoluminescence excitation spectra recorded at emission wavelengths of 529 nm (QDG), 577 nm (DyeG), 661 nm (QDR), and 712 nm (DyeR). Here, the deviations of the fixed emission wavelengths from the maximum emission wavelengths are chosen to avoid contributions from scattered excitation light, but do not alter the results. All beads can be excited at 488 nm which is subsequently used for the LT-FCM studies. (**b**) Photoluminescence emission spectra recorded at excitation wavelengths of 425 nm (QDs) or 488 nm (dyes). In the case of the QDs, the solid lines represent Gaussian fits to the data points on the energy scale. All codes emit in the spectral region >530 nm as required for detection with the LT-FCM setup.

The photoluminescence spectra of the differently encoded beads are shown in Fig. 1.

The photoluminescence excitation spectra summarized in Fig. 1(a) show the typical characteristics of organic dyes and QDs, i.e., absorption bands in a confined wavelength region and the steadily increasing absorption at wavelengths below the optical bandgap enabling a quasi-free choice of the excitation wavelength. The dye-loaded beads reveal fluorescence excitation maxima at 533 nm (DyeG) and around 630 nm (DyeR). All samples can be excited at 488 nm as needed for measurements with our LT-FCM setup. The emission spectra of the corresponding bead ensembles are displayed in Fig. 1(b). The emission spectrum of the Rh6G-stained PMMA beads (DyeG) with a maximum at around 550 nm resembles literature reports on this system^51^, the emission maximum of Red5 (DyeR)-loaded beads is located at around 690 nm. The emission spectra of the QD-encoded beads QDG and QGR are roughly Gaussian shaped as revealed by the good match of the measured spectra and Gaussian fits on an energy scale (see Fig. 1(b), solid lines). From these fits, we determined the emission maxima of QDG and QDR to 508 nm (2.44 eV) and 648 nm (1.91 eV), respectively. The deviation from the nominal emission maximum found could be possibly explained by the different microenvironment faced by the QDs upon incorporation into the microbead polyelectrolyte shell. Based upon the Gaussian fits of the QD emission profiles, we extracted full widths at half maximum (FWHM) of 199 meV and 86 meV for QDG and QDR, respectively. This distribution of emission energies is attributed to size variations of the QDs. Based on the position of their spectral emission maxima, the differently encoded beads can be divided into two ‘green’ codes (DyeG and QDG) and two ‘red’ codes (DyeR and QDR) for the subsequent LT-FCM studies.

**Figure 2:**
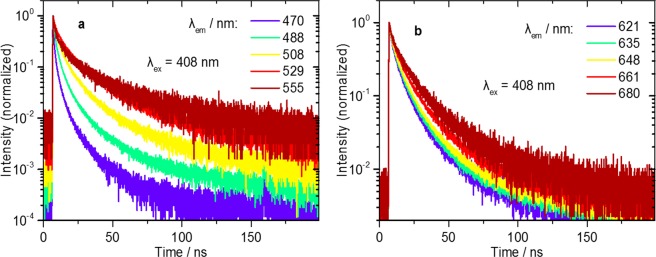
Photoluminescence decay curves of ensembles of QD-loaded beads. (**a**) Decay curves of QDG, and (**b**) decay curves of QDR.

Prior to the LT-FCM studies, time-resolved luminescence studies were performed with ensembles of QD-encoded beads. In order to examine possible wavelength dependencies of the photoluminescence decay dynamics, the luminescence intensity decays were measured at several positions across the emission band. Figure 2 shows the photoluminescence decay curves of the QD-encoded beads determined with an excitation wavelength of 408 nm. This figure reveals a pronounced and a moderate dependence of the decay kinetics on the emission wavelength for QDG and QDR, respectively, which seems to mainly originate from the size distribution of the incorporated QDs.

As highlighted in Fig. 2, the luminescence decays of both types of QD-encoded beads show a multi-exponential behaviour, with the mean lifetime increasing with increasing emission wavelength. Besides background contributions^21^, we tentatively attribute this observation to the presence of QDs with slightly different optical properties within the beads. It needs to be kept in mind that each QD ensemble contains nanocrystals of different size and most likely also slightly different surface chemistries, resulting in slightly different energy bands and emission maxima (see Fig. S3 of the ESI). At shorter emission wavelengths, the contribution of smaller-sized QDs to the observed photoluminescence is more pronounced and the mean lifetime is thus shorter than the lifetime obtained at longer wavelengths due to the direct relation between the size of the QDs and their radiative lifetime^52,53^. The radiative lifetime of such II/VI QDs is about 20–40 ns^52–54^, and the longest components in the luminescence decay kinetics of the QDs used here are within this time range. This suggests, that despite some QDs having a high photoluminescence quantum yield, the average lifetimes of both QD ensembles are, however, shortened due to a large proportion of QDs with a lower photoluminescence quantum yield^55–57^. As previously mentioned, the more pronounced emission wavelength dependence of the decay kinetics of QDG shown in Fig. 2 reflects their broader size distribution in accordance with the broadened emission spectra highlighted in Fig. 1(b). It should be noted here, that for LT-FCM single-exponential decays of the encoding luminophores are preferred, but not mandatory.

**Figure 3:**
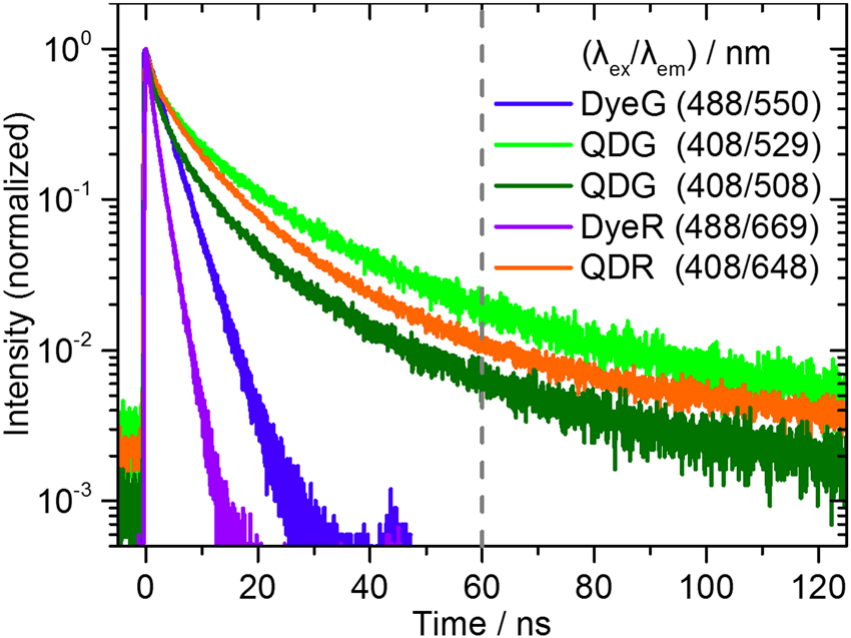
Photoluminescence decay curves of ensembles of luminophore-encoded beads suspended in water. The excitation and emission wavelengths used for the measurements are indicated in the figure. For QDG, two curves are displayed to point out the wavelength dependence of its decay kinetics.

The photoluminescence decay curves of all four lifetime codes are displayed in Fig. 3 and the corresponding fluorescence lifetimes obtained as intensity-weighted average values from multi-exponential decay fits of the measured data are summarized in Table 1. These lifetime values are only rough values due to the strong variation of the decay kinetics across the emission spectra. These data are used to model the integral LT-FCM measurements, where the selection of the detected photons was done using a 530 longpass filter. Figure 3 also underlines the significantly different decay kinetics of the dye- and QD-encoded beads DyeG, DyeR, QDG, and QDR. As expected, the luminescence lifetimes of the dye-encoded beads DyeG and DyeR are much shorter than those of the QD-based samples QDG and QDR, which are well-known for their longer fluorescence lifetimes^58^. The slightly multi-exponential decay behavior even of the dye-encoded beads is ascribed to the inhomogeneous microenvironment of the immobilized dye molecules within the beads.

**Table 1:** Optical properties of the studied bead populations including measurement parameters, bead diameter D, ensemble (reference) lifetimes, and FLIM lifetimes as well as lifetimes from the LT-FCM measurements.

Code	Luminophore	*Diameter D*/μm	*λ*_ex_/nm	*λ*_det_/nm	τ_int_/ns		
					ensemble	FLIM	LT-FCM
DyeG	Rh6G	5.4 ± 0.5	488	550	3.5		
			485	*525LP*		3.3 ± 0.04	
			488	*530LP*			9.4 ± 0.9
QDG	CdSe/ZnS	6.2 ± 0.6	408	508*)	13.5		
			485	*525LP*		8.7 ± 0.2	
			488	*530LP*			21 ± 3
DyeR	Red5	6.5 ± 0.5	488	669	1.6		
			485	*525LP*		1.9 ± 0.05	
			488	*530LP*			10.1 ± 1
QDR	CdSe/CdS/ZnS	6.2 ± 0.6	408	648*)	15.7		
			485	*525LP*		9.8 ± 0.01	
			488	*530LP*			24 ± 3

The ensemble and the FLIM-based lifetimes were calculated as intensity-weighted mean lifetimes from multiexponential decay fits. FLIM-based lifetimes were obtained from individual measurements of each lifetime code.

^*^Measurement at the emission maximum.

According to Fig. 3, the set of the four different lifetime codes can be separated into two groups: organic dye-based beads DyeG and DyeR with short excited state lifetimes <10 ns and QDs-based beads QDG and QDR with excited state lifetimes >10 ns. Considering also the spectral fluorescence properties shown in Fig. 1(b), the four samples are characterized by a unique combination of different emission maxima (‘green’ and ‘red’) and lifetimes (‘short’ and ‘long’): DyeG (‘green’ & ‘short’), QDG (‘green’ & ‘long’), DyeR (‘red’ & ‘short’), QDR (‘red’ & ‘long’). Thus, these four luminophore-encoded bead populations are suitable for combined spectral and temporal multiplexing with our LT-FCM setup. The resulting lifetime codes range from about 2 ns for DyeR up to about 16 ns in the case of QDG. All optical properties of the lifetime-encoded beads, including luminescence lifetimes obtained as intensity-weighted average values from multi-exponential decay fits to the data are summarized in Table 1.

### Optical properties of single microbeads - Confocal laser scanning microscopy (CLSM) and fluorescence lifetime imaging microscopy (FLIM)

**Figure 4:**
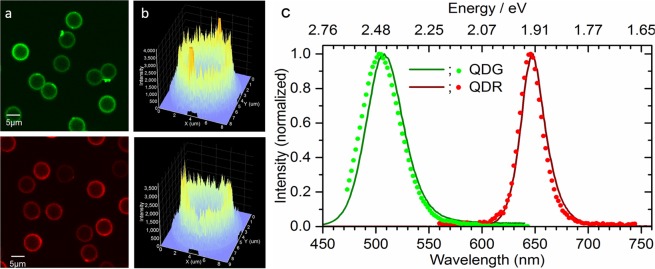
Confocal laser scanning microscopy studies of QD-encoded polymer beads QDG (upper panel (a,b)) and QDR (lower panel (a,b)), excitation wavelength 458 nm. (**a**) False colour fluorescence intensity images; (**b**) Exemplary intensity profiles of selected beads; (**c**) Spatially resolved photoluminescence spectra (spectrally uncorrected) of representative single QD-loaded beads (symbols) compared to the respective spectrally corrected luminescence spectra derived from ensemble measurements (solid lines).

CLSM was used for the characterization of the QD-loaded beads QDG and QDR on the single bead level. The resulting CLSM images are shown in Fig. 4 (QDG: left upper panel; QDR: left lower panel). As to be expected for beads decorated with QD-LbL shells, the photoluminescence intensity shows a corona-like spatial distribution without emission from the bead cores. This is also apparent from the respective luminescence intensity profiles shown in Fig. 4(b). The luminescence images reveal bead-to-bead variations in signal intensities, which indicate different numbers of QDs within the LbL shell of the individual beads. This suggests that for the chosen encoding strategy, the QD incorporation efficiency is prone to variations from bead to bead. The emission spectra of representative single beads obtained with CLSM are displayed in Fig. 4(c). The spectral position of the emission maxima derived for QDG and QDR are 503 nm and 645 nm, respectively. Both values are in reasonable agreement with the ensemble measurements shown in Fig. 1, that are, however, more reliable due to more sophisticated spectral correction procedures used for ensemble measurement with the calibrated fluorometer^59^. Obviously, the number of encoding QDs in the polymer particles is large enough to average out effects originating from the QD size distribution.

**Figure 5:**
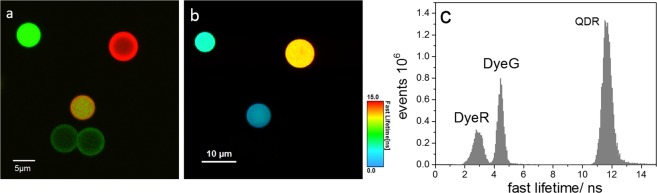
CLSM and FLIM images of dye- and QD-loaded beads exhibiting short and (relatively) long luminescence lifetimes. (**a**) CLSM image in two different spectral channels (green: 500–600 nm, red: 600–700 nm) with false colour encoding (excitation wavelength 488 nm). (**b**) FLIM image of the same beads showing three of the four different lifetime codes. The QDG code could not be displayed because of a limited emission intensity in the detection window used (measurement parameters see Table 1). (**c**) Well separated luminescence lifetime distributions of three of the four dye- and QD-encoded beads obtained from the Fast-FLIM image^60^ shown in (**b**).

Figure 5(a) shows a CLSM image of luminophore-encoded beads containing particles from all four populations measured in two different spectral channels. All dye-loaded beads exhibit a homogeneous dye distribution as to be expected from the staining procedure used. This procedure leads only to very small bead-to-bead intensity variations (data not shown), indicating comparable loading concentrations for the dye-encoded beads of one lifetime code. The CLSM image in Fig. 5(a) also highlights the different luminescence intensities arising from the excitation of all samples at 488 nm. In this case, the luminescence intensity from the QD-encoded beads QDG is much lower than those resulting for the other three bead populations.

Luminescence lifetime information on QD-decorated and dye-loaded beads at the single-particle level was obtained by FLIM. The FLIM image of mixed bead populations displayed in Fig. 5(b) represents a visualization of three of our four lifetime-encoded bead types. The QDG code could not be displayed in the image of the mixed beads because of limited emission intensity in the detection window used.

**Figure 6:**
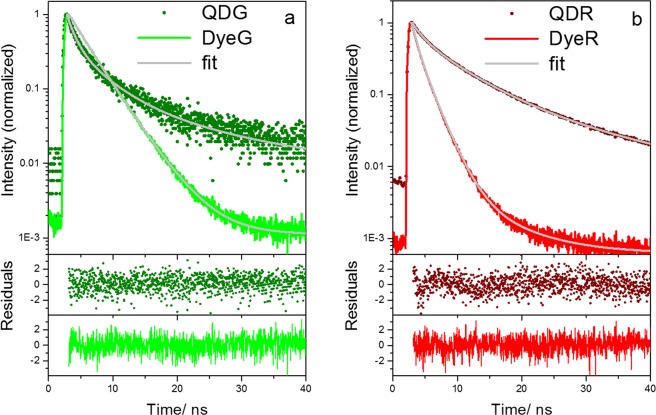
Comparison of the multi-exponential luminescence decay kinetics detected in the ‘green’ (**a**) and the ‘red’ (**b**) channel of the FLIM setup revealing the fluorescence decays and tri-exponential fits (grey lines) of exemplarily chosen representative individual dye-stained PMMA beads (solid lines) and QD-decorated beads (solid symbols). The bottom panels show the deviation between recorded and fitted data.

Representative luminescence decay curves of single beads obtained by FLIM are shown in Fig. 6. The intensity-weighted mean lifetimes of the individual beads were calculated from fits of the multi-exponential decay curves. Even though the photon count number and dynamic range are reduced in comparison to the ensemble measurements shown in Fig. 3, the decay kinetics of single beads belonging to the four different bead populations can be still clearly distinguished.

### Time-resolved lifetime flow cytometry (LT-FCM)

The suitability of the four bead populations characterized in the previous sections as potential encoding beads for a multiplexing strategy relying on both spectral characteristics and luminescence decay kinetics are subsequently assessed using an excitation wavelength of 488 nm and the novel compact LT-FCM instrument. The photoluminescence decays were detected with a 530 nm longpass filter in front of the photon counting detector used in the LT-FCM setup. Additionally, steady-state intensities were acquired with a (520 ± 14) nm bandpass filter (‘green’ channel).

**Figure 7:**
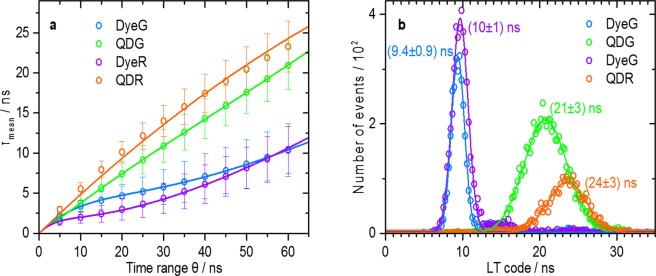
Lifetime code detection in flow cytometry. (**a**) Dependence of the observed mean lifetime on the chosen time range *θ*. Solid lines represent fits of Eq. (2) to the data points. (**b**) Luminescence lifetime distributions obtained with LT-FCM with a time range of 60 ns. Solid lines are Gaussian fits of the distributions.

For lifetime determination in LT-FCM, the straightforward adaption of the integration time range $\theta$ in Eq. (1) can be used to drastically improve the discrimination capabilities^21^. The dependence of the mean lifetime ${\tau }_{{\rm{mean}}}$ of single-bead measurements on the time range $\theta$ measured with our LT-FCM setup is shown Fig. 7(a). A steady increase of the obtained mean lifetime values with increasing $\theta$ is observed. Moreover, the two short lifetime codes resulting for the dye-encoded beads DyeG and DyeR converge to a common value for $\theta \approx 55\,{\rm{ns}}$. The dependence of the lifetime on the time range can be explained by introducing a model function for the fluorescence decay in Eq. (1)^21^. In this case, we chose a general expression for multiexponential decays with amplitudes *A*_*j*_ and constant background $B$ and obtained Eq. (2) for the theoretical dependence of ${\tau }_{{\rm{mean}}}$ on $\theta$.

2 $${\tau }_{{\rm{mean}},{\rm{bg}}}=\frac{{\sum }_{j}({A}_{j}{\tau }_{j}[{\tau }_{j}-(\theta +{\tau }_{j}){e}^{-\frac{\theta }{{\tau }_{j}}}])+\frac{B}{2}{\theta }^{2}}{{\sum }_{j}({A}_{j}{\tau }_{j}[1-{e}^{-\frac{\theta }{{\tau }_{j}}}])+B\theta }$$

The fits of the measured data with Eq. (2) in Fig. 7(a) show a perfect agreement for DyeG and DyeR (mono-exponential) and QDG (bi-exponential). The agreement for QDR is moderate. The steady increase of the lifetime obtained for increasing $\theta$ is caused by background counts that are represented by the parameter $B$ in Eq. (2). These background counts gain in importance the larger the time range $\theta$ becomes^21^. The LT code values are only meant to be used for discrimination or detection of differences between the four bead population but not for an absolute characterization of the decay characteristics of the samples. To achieve the largest separation and hence best distinction between the two groups of codes with short (DyeG and DyeR) and long (QDG and QDR) lifetimes, it is advantageous to choose the largest possible time range of $\theta =60\,{\rm{ns}}$, even though this results in a strong overlap of the two short lifetime codes.

The lifetime code distributions obtained with the optimized time range of 60 ns are shown in Fig. 7(b). Here, the measured lifetime codes range from about 10 ns up to more than 20 ns. The lifetime codes are clearly separated into two groups with short and long lifetimes as was aimed for. The two short and the two long-lived codes, however, considerably overlap. Figure 7(a,b) also illustrate the fact that the LT-FCM setup in its current state is only suitable for the distinction of different lifetime codes and not for measuring actual lifetime values. The mean lifetime values measured in LT-FCM exceed the ensemble lifetimes summarized in Table 1 and strongly vary with the chosen time range $\theta$. The luminescence decay curves of the QD-encoded samples in Fig. 2 suggest that the contribution of the long-lived decay components dominates the decay dynamics at longer times after excitation (see Fig. 2). The slower decay components can be hardly distinguished from background counts and therefore the lifetimes calculated by Eq. (1) lead to a systematic overestimation of the lifetime values.

As follows from Fig. 7(b), the lifetime code distributions of the dye-stained beads are narrower than those of the QD-loaded beads. As every bead contains a large number of QDs^40^, it is rather unlikely that the decay dynamics differ from bead to bead. The broadened LT code distribution in the case of QDR possibly originates from a technical issue. Longer lifetimes obtained for beads with higher intensities can be caused by detector saturation effects^21^. In the case of detector saturation, the measured lifetimes appear to be prolongated. Thus, QD-loaded beads with lower luminescence intensities contribute to the measured decay kinetics with shorter lifetimes than brighter QD-loaded beads that can lead to detector saturation resulting in artificially enhanced lifetimes. A wider range of fluorescence intensities, as observed for these samples by CLSM, Fig. 4(a), could therefore translate into a wider range of measured lifetimes in LT-FCM. The larger width of the lifetime distribution of code QDG is probably related to the relatively low photon count number introducing a larger variance of the obtained values.

**Figure 8:**
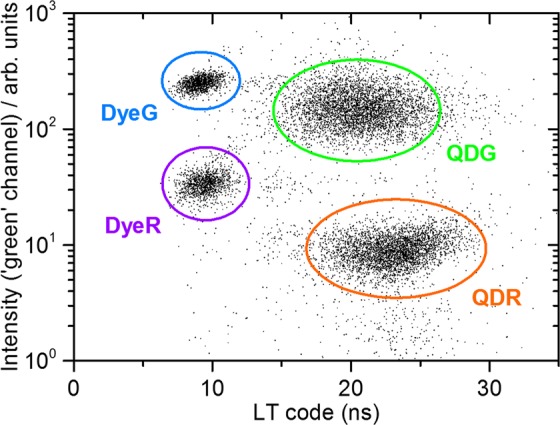
Dot-plot correlating the lifetime codes and the fluorescence intensity in the ‘green’ channel. The four different bead populations are clearly distinguishable. FL1 intensities of 37 ± 13 (1 < FL1 < 200) for DyeR, 10 ± 5 (0 < FL1 < 100) for QDR, 272 ± 27 (100 < FL1 < 400) for DyeG, 149 ± 63 (1 < FL1 < 600) for QDG were obtained from statistical evaluation. For statistics (standard deviations) regarding lifetime data, refer to Fig. 7(b).

Using the additional ‘green’, (520 ± 14) nm, channel to exploit the spectral differences between the codes, the dot-plot displayed in Fig. 8 is obtained. Here, four distinct populations related to the four codes can be clearly distinguished. Dye-based codes with short lifetimes can be clearly separated from the QD-based codes with longer lifetimes and the ‘green’ codes can be distinguished from the ‘red’ codes based on their different intensities in the ‘green’ channel.

## Conclusion and Outlook

In summary, we evaluated the potential of a set of four luminescent polymer bead samples, encoded with two organic dyes and two different CdSe-based core/shell quantum dots (QDs) for a new multiplexing approach using a unique prototype of a time-resolved flow cytometer setup, equipped with a single excitation light source and several detectors. In our studies, one detector was used as the ‚green‘ channel to exploit spectral differences of the codes based on their intensity values. An additional lifetime detector operated in the photon counting mode was employed to determine the luminescence lifetimes.

Our results confirm that the combination of dye-stained polymer microparticles, presenting bead populations with relatively short lifetimes, and II/VI-QD-based bead populations with longer lifetimes exceeding 10 ns, is well suited for the intended tempo-spectral multiplexing scheme. With four lifetime codes and the lifetime flow cytometer, we could demonstrate the straightforward combination of spectral and temporal multiplexing. The four codes could be clearly distinguished by means of their unique combination of colour (‘green’/‘red’) and lifetime (‘short’/‘long’). In addition, based upon this study, requirements on encoded beads and encoding luminophores for tempo-spectral multiplexing in flow cytometry with inexpensive instrumentation could be derived. Obviously important for the preparation of the next generation of encoded beads are particles of comparable fluorescence intensities whereas requirements on the decay kinetics are not as strict as revealed by the multi-exponentially decaying QDs. More work is, however, required to further improve the LT-FCM setup and to gain control over intensity-dependent effects on lifetime encoding to ensure reproducibility.

Overall, we could show that by including a single spectrally integrating detector for photon counting with nanosecond time resolution, another dimension in parameter space can be added to flow cytometry measurements in the time-domain. This approach paves the way for new luminescence lifetime-based encoding strategies in flow cytometry meeting the requirements of increasingly complex analytical research.

## Supplementary information

Supplementary Information
