# Supplementary material for: Tempo-spectral multiplexing in flow cytometry with lifetime detection using QD-encoded polymer beads
Source: Sci Rep. 2020 Jan 20;10:653. doi: 10.1038/s41598-019-56938-2 (PMC6971033; doi:10.1038/s41598-019-56938-2)
Supplement: Supplementary file 1 — Supplementary Information [file 41598_2019_56938_MOESM1_ESM.pdf]

Supplementary Information

Tempo-spectral multiplexing in flow cytometry with lifetime detection using QD-encoded polymer beads

Daniel Kage[1,2], Katrin Hoffmann[1], Galina Nifontova[3], Victor Krivenkov[3], Alyona Sukhanova[4], Igor Nabiev[3,4], and Ute Resch-Genger[1]*

[1]Federal Institute for Materials Research and Testing (BAM), Biophotonics Division 1.2, Richard-Willstätter-Str. 11, D-12489, Berlin, Germany

[2]Department of Physics, Humboldt-Universität zu Berlin, Newtonstr. 15, D-12489, Berlin, Germany

[3]Laboratory of Nano-bioengineering, National Research Nuclear University MEPhI (Moscow Engineering Physics Institute), 115409 Moscow, Russian Federation

[4]Laboratoire de Recherche en Nanosciences, LRN-EA4682, Université de Reims Champagne-Ardenne, 51100 Reims, France

*e-mail: ute.resch@bam.de

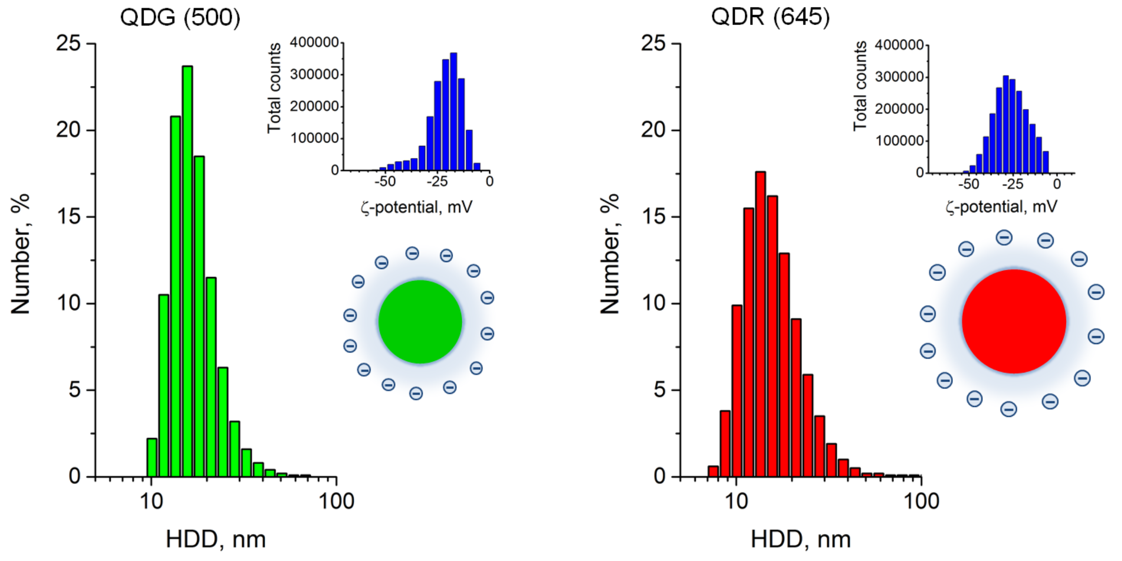

Figure S1. Colloid characteristics of QDG and QDR used in the study: hydrodynamic diameter (HDD) and ζ-potential distributions.

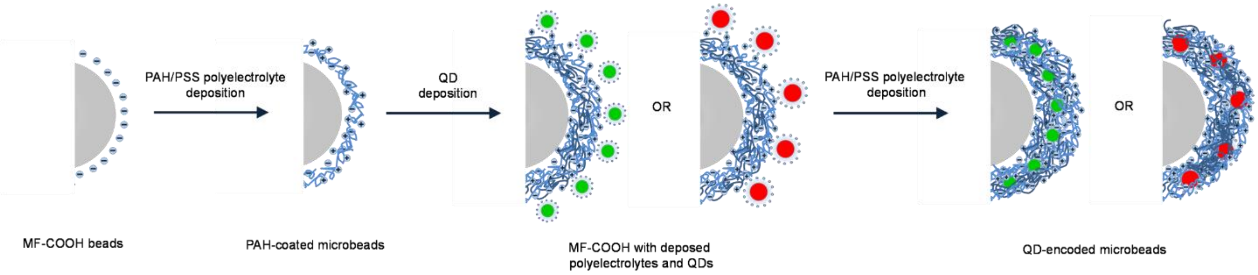

Figure S2. Scheme of the layer-by-layer (LbL) preparation[1,2] of QD-encoded polymer beads performed with QDG (QD500) and QDR (QD645) (shown in profile).

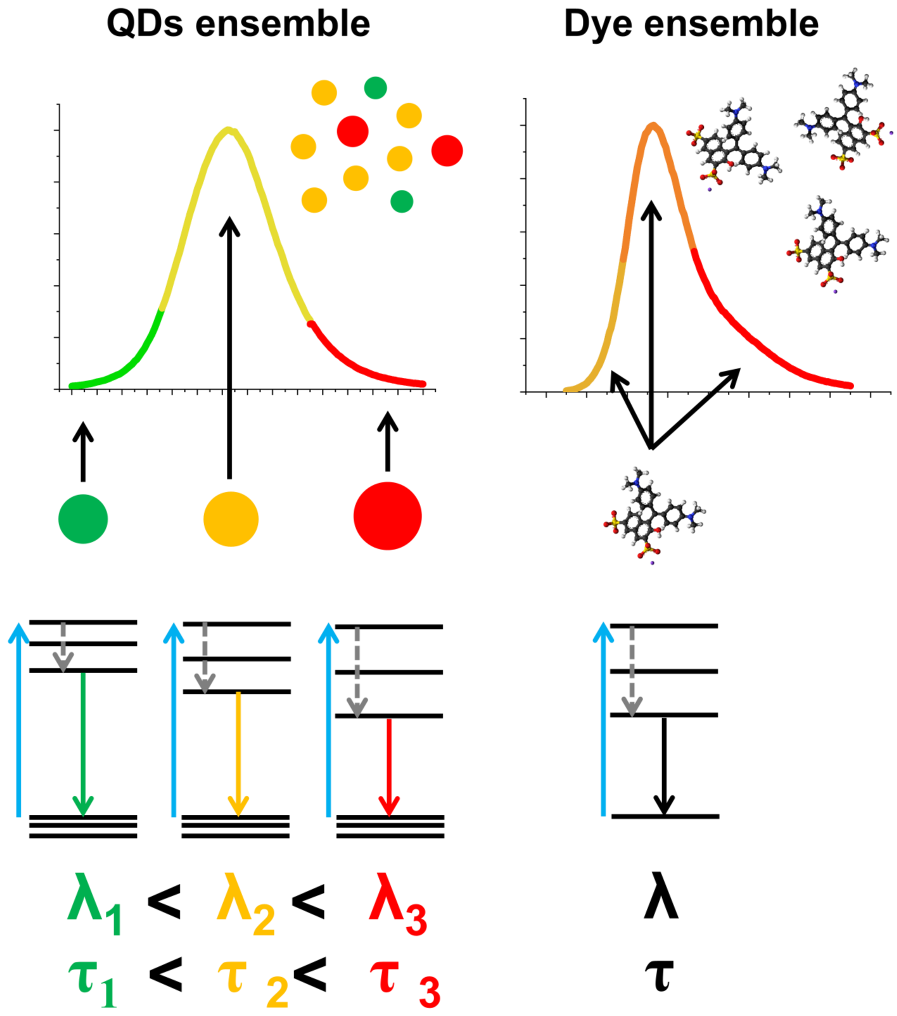

Figure S3. Very basic sketches of the mechanistic pathway of the decay profiles of QDs ensembles used in the LbL coating of lifetime-encoded beads (left) and of ensembles of dye molecules (right) incorporated in polymer beads.

References

1 Bilan, R., Nabiev, I. & Sukhanova, A. Quantum Dot-Based Nanotools for Bioimaging, Diagnostics, and Drug Delivery. *Chembiochem* **17**, 2103-2114, doi:10.1002/cbic.201600357 (2016).

2 Bilan, R. S. *et al.* Engineering of Optically Encoded Microbeads with FRET-Free Spatially Separated Quantum-Dot Layers for Multiplexed Assays. *Chemphyschem* **18**, 970-979, doi:10.1002/cphc.201601274 (2017).
